# Understanding Zika Virus Stability and Developing a Chimeric Vaccine through Functional Analysis

**DOI:** 10.1128/mBio.02134-16

**Published:** 2017-02-07

**Authors:** Xuping Xie, Yujiao Yang, Antonio E. Muruato, Jing Zou, Chao Shan, Bruno T. D. Nunes, Daniele B. A. Medeiros, Pedro F. C. Vasconcelos, Scott C. Weaver, Shannan L. Rossi, Pei-Yong Shi

**Affiliations:** a Department of Biochemistry and Molecular Biology, University of Texas Medical Branch, Galveston, Texas, USA; b College of Animal Science and Technology, Southwest University, Chongqing, China; c Institute for Human Infections and Immunity, University of Texas Medical Branch, Galveston, Texas, USA; d Institute for Translational Science, University of Texas Medical Branch, Galveston, Texas, USA; e Seção de Arbovirologia e Febres Hemorrágicas, Instituto Evandro Chagas, Ministério da Saúde, Ananindeua, Pará, Brazil; f Department of Microbiology and Immunology, University of Texas Medical Branch, Galveston, Texas, USA; g Sealy Center for Vaccine Development, University of Texas Medical Branch, Galveston, Texas, USA; h Center for Biodefense and Emerging Infectious Diseases, University of Texas Medical Branch, Galveston, Texas, USA; i Department of Pathology, University of Texas Medical Branch, Galveston, Texas, USA; j Sealy Center for Structural Biology & Molecular Biophysics, University of Texas Medical Branch, Galveston, Texas, USA; k Department of Pharmacology & Toxicology, University of Texas Medical Branch, Galveston, Texas, USA; Columbia University

## Abstract

Compared with other flaviviruses, Zika virus (ZIKV) is uniquely associated with congenital diseases in pregnant women. One recent study reported that (i) ZIKV has higher thermostability than dengue virus (DENV [a flavivirus closely related to ZIKV]), which might contribute to the disease outcome; (ii) the higher thermostability of ZIKV could arise from an extended loop structure in domain III of the viral envelope (E) protein and an extra hydrogen-bond interaction between E molecules (V. A. Kostyuchenko, E. X. Y. Lim, S. Zhang, G. Fibriansah, T.-S. Ng, J. S. G. Ooi, J. Shi, and S.-M. Lok, Nature 533:425–428, 2016, https://doi.org/10.1038/nature17994). Here we report the functional analysis of the structural information in the context of complete ZIKV and DENV-2 virions. Swapping the prM-E genes between ZIKV and DENV-2 switched the thermostability of the chimeric viruses, identifying the prM-E proteins as the major determinants for virion thermostability. Shortening the extended loop of the E protein by 1 amino acid was lethal for ZIKV assembly/release. Mutations (Q350I and T351V) that abolished the extra hydrogen-bond interaction between the E proteins did not reduce ZIKV thermostability, indicating that the extra interaction does not increase the thermostability. Interestingly, mutant T351V was attenuated in A129 mice defective in type I interferon receptors, even though the virus retained the wild-type thermostability. Furthermore, we found that a chimeric ZIKV with the DENV-2 prM-E and a chimeric DENV-2 with the ZIKV prM-E were highly attenuated in A129 mice; these chimeric viruses were highly immunogenic and protective against DENV-2 and ZIKV challenge, respectively. These results indicate the potential of these chimeric viruses for vaccine development.

## IMPORTANCE

Analysis of a recently observed high-resolution structure of ZIKV led to a hypothesis that its unusual stability may contribute to the associated, unique disease outcomes. Here we performed a functional analysis to demonstrate that viral prM-E genes are the main determinants for the high stability of ZIKV. The extra hydrogen-bond interaction (observed in the high-resolution structure) between ZIKV E proteins did not enhance virion stability, whereas the extended loop of E protein (CD loop in domain III) was essential for ZIKV assembly. More importantly, we found that a chimeric ZIKV with DENV-2 prM-E genes and a chimeric DENV-2 with ZIKV prM-E genes were highly attenuated in A129 mice. Mice immunized with these chimeric viruses generated robust neutralizing antibody responses and were fully protected from DENV-2 and ZIKV challenge, respectively, indicating that these chimeric viruses could be further developed as vaccine candidates.

## INTRODUCTION

Zika virus (ZIKV) is a mosquito-borne member of the genus *Flavivirus* within the family *Flaviviridae*. Many flaviviruses, such as the four serotypes of dengue virus (DENV-1 to DENV-4), yellow fever virus (YFV), West Nile virus (WNV), Japanese encephalitis virus (JEV), and tick-borne encephalitis virus (TBEV), are significant human pathogens. ZIKV was first isolated from a sentinel rhesus macaque in 1947 in the Zika Forest of Uganda (1). During the next six decades, ZIKV was detected predominantly in sylvatic transmission cycles between nonhuman primates and arboreal mosquitoes and was rarely associated with human disease (2). Since 2007, ZIKV has caused outbreaks/epidemics across islands in the Pacific and Americas, including on Yap Island in Micronesia and subsequently in French Polynesia, and, most recently, explosive epidemics in the Americas (2). According to the recent report from the World Health Organization (http://www.who.int/emergencies/zika-virus/situation-report/6-october-2016/en/), about 73 countries and territories have reported evidence of autochthonous ZIKV transmission.

Most human ZIKV infections are asymptomatic. Symptomatic infections show “dengue-like” manifestations, such as fever, headaches, lethargy, conjunctivitis, rash, arthralgia, and myalgia. Only recently was it recognized that ZIKV infection can cause severe diseases, including neurotropic Guillain-Barre syndrome and congenital microcephaly (3). Besides viremia, ZIKV can be detected in urine (4), saliva (5), and semen (6) during human infections. Although ZIKV is mainly transmitted by *Aedes aegypti* mosquitoes (7), recent evidence shows that direct, interhuman transmission can also occur sexually (6, 8) or vertically (9) or through blood transfusion and organ transplantation (10). Better understanding of the mechanisms of ZIKV replication, pathogenesis, and transmission would facilitate vaccine and antiviral development.

Flaviviruses have a positive single-strand RNA genome of approximately 11,000 nucleotides in length. The genome contains a 5′ untranslated region (UTR), a long open-reading frame (ORF), and a 3′ UTR. The ORF encodes three structural (capsid [C], precursor membrane [prM], and envelope [E]) and seven nonstructural (NS1, NS2A, NS2B, NS3, NS4A, NS4B, and NS5) proteins. Along with genomic RNA, the structural proteins form viral particles. The nonstructural proteins participate in viral replication, virion assembly, and evasion of host immune response (11). Two recent cryo-electron microscopy (cryo-EM) studies showed that the mature ZIKV structure (12, 13) is similar overall to those of DENV (14) and WNV (15). ZIKV contains an interior nucleocapsid formed by multiple copies of C protein and viral genomic RNA as well as an icosahedral shell consisting of 180 copies of E and M proteins (or prM) embedded in a host-derived lipid bilayer (12, 13). The E protein is involved in receptor binding and membrane fusion. Compared with DENV, based on virion imaging, two distinct structural features were reported for the ZIKV E protein, including an extended glycan loop (13) and a hydrogen-bond interaction between residues Q350 and T351 in an extended CD loop at domain III around the 5-fold vertex (12). These differences were hypothesized to account for cellular tropism and virion stability, leading to distinct pathogenesis during ZIKV infection (12, 13). Using a WNV replicon-based virus-like particle (VLP) system, Goo and colleagues recently showed that (i) mutations of Q350A and T351A did not alter the thermostability of ZIKV structural-protein-packaged WNV VLPs and (ii) high thermostability is not unique to ZIKV because WNV possessed an even higher level of thermostability (16). It remains to be determined whether the same mutations affect the thermostability of wild-type (WT) ZIKV and, more importantly, whether the thermostability affects viral pathogenesis *in vivo*.

In this study, we constructed chimeric ZIKV and DENV-2 viruses containing heterologous prM-E genes. Using these chimeras, we demonstrate that prM-E genes are the major determinants of virion thermostability. The prM-E chimeric viruses (CHV) were attenuated *in vivo*. Mice immunized with the chimeric viruses were fully protected from WT ZIKV and DENV challenges. In addition, our mutagenesis results demonstrated that the extra hydrogen-bond interaction in the E proteins (observed in the recent high-resolution structure) does not contribute to the stability of ZIKV.

## RESULTS

### Generation and characterization of chimeric ZIKV and DENV-2.

**FIG 1:**
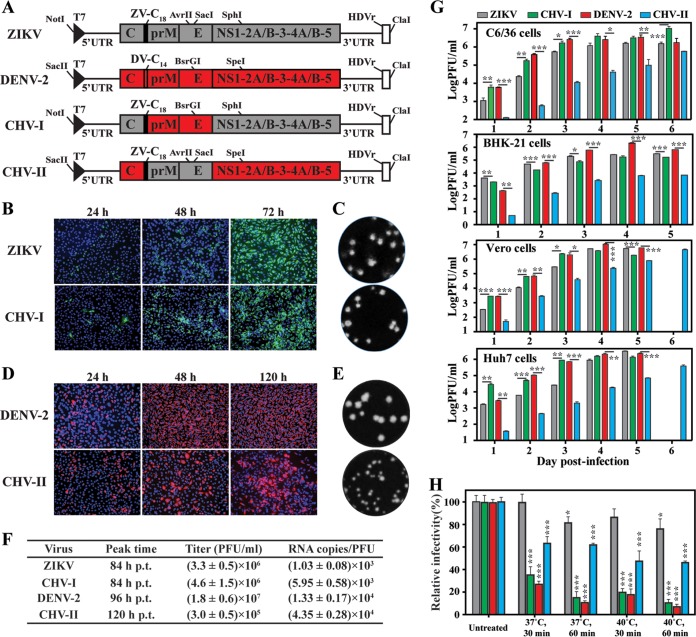
Generation and characterization of ZIKV and DENV-2 chimeric viruses. (A) Schematics of construction of ZIKV and DENV-2 chimeric viruses. ZV-C_18_, anchor C of ZIKV; DV-C_14_, anchor C of DENV-2. Restriction enzyme sites used for cloning are indicated. The drawing is not to scale. (B) Immunofluorescence assay (IFA). Vero cells were electroporated with equal amounts of ZIKV or CHV-1 RNAs. Mouse MAb 4G2 and goat anti-mouse IgG conjugated with Alexa Fluor 488 were used as primary and secondary antibodies. Nuclei were stained using DAPI. (C) Plaque morphologies of ZIKV and CHV-1. Plaques were developed on a Vero cell monolayer after 4.5 days of infection. (D) IFA analysis of E protein expression in BHK-21 cells transfected with DENV-2 and CHV-II RNAs. E protein was examined by IFA using 4G2 and goat anti-mouse IgG conjugated with Alex Flor 568. (E) Plaque morphologies of DENV-2 and CHV-II viruses. Plaques were developed on a BHK-21 cell monolayer after 4 days of infection. (F) Virus titers and RNA copy number/PFU ratios. From 24 to 120 h p.t., supernatants were harvested at every 12 h. Levels of infectious virions (quantified in PFU per milliliter) in the culture fluids were determined by plaque assay. The copies of extracellular viral RNA at the peak titer time (RNA copies per milliliter) were quantified by qRT-PCR. (G) Virus growth kinetics. C6/36, BHK-21, Vero, and Huh7 cells were infected with ZIKV, DENV-2, CHV-I, and CHV-II at an MOI of 0.01. Extracellular infectious virions were quantified by plaque assay with a limit of detection of 10 PFU/ml. Data show mean titers from results of three independent experiments. A multiple *t* test was performed to analyze the statistic differences in the titers between ZIKV and CHV-I or the titers between DENV-2 and CHV-II at the indicated time points. *, *P* < 0.05 (significant); **, *P* < 0.01 (very significant); ***, *P* < 0.001 (extremely significant). (H) Thermostability analysis of chimeric ZIKV and DENV-2. Data indicate the means of results from three independent experiments. One-way analysis of variance (ANOVA) was performed to analyze the statistical differences between each treatment group and the corresponding untreated group.

We made two chimeric viruses using both ZIKV and DENV infectious cDNA clones (Fig. 1A). Chimeric virus I (CHV-I) contained a substitution of DENV-2 prM-E for ZIKV prM-E genes in the backbone of the ZIKV genome. CHV-II contained a substitution of ZIKV C_18_-prM-E for DENV-2 C_14_-prM-E genes in the backbone of the DENV-2 genome; C_18_ and C_14_ represent the 18 and 14 amino acids of anchor C from ZIKV and DENV-2, respectively. To recover recombinant viruses, we electroporated ZIKV and CHV-1 genomic RNAs into Vero cells (commonly used to make recombinant ZIKV virus) and electroporated DENV-2 and CHV-II genomic RNAs into BHK-21 cells (commonly used to prepare recombinant DENV virus). Both the WT ZIKV and CHV-I RNAs generated increasing numbers of E protein-expressing cells from 24 to 72 h posttransfection (p.t.; Fig. 1B). Similar plaque morphologies were observed for WT ZIKV and CHV-I (Fig. 1C). Comparable amounts of infectious viruses were produced from the ZIKV and CHV-I RNA-transfected cells, with peak titers of 3 to 5 × 10^6^ PFU/ml at 84 h p.t. (Fig. 1F). In contrast, compared with DENV-2 RNA, CHV-II RNA was delayed in the increase of the level of E-expression cells after transfection (Fig. 1D). The recovered CHV-II generated smaller plaques than the WT DENV-2 (Fig. 1E). The CHV-II RNA-transfected cells were delayed by 24 h in producing the peak viral titer compared with the WT DENV-2 RNA-transfected cells (Fig. 1F). Furthermore, the peak titer of CHV-II was 60-fold lower than that of WT DENV-2 (Fig. 1F). It is worth pointing out that plaque assays for ZIKV and CHV-I could be performed on Vero or BHK-21 cells and that similar viral titers were obtained using Vero and BHK-21 cells (data not shown). However, the plaque assays for DENV-2 and CHV-II were performed on BHK-21 cells because DENV-2 did not form visible plaques on Vero cells after crystal violet staining.

To examine the effect of chimeric prM-E proteins on virus infectivity, we determined the RNA copy number/PFU ratio for each of the recovered viruses. The extracellular viral RNA copy numbers represented total numbers of viruses (including both infectious and noninfectious virions) released into the culture fluids. The PFU numbers indicated the amounts of infectious virions. Interestingly, the WT ZIKV showed an almost 10-fold-lower RNA copy number/PFU ratio than the WT DENV-2 (Fig. 1F), suggesting that ZIKV was more efficient in packaging viral RNA into infectious virions than DENV-2 under our experimental conditions. In addition, CHV-I and CHV-II showed over 5-fold-higher and 3-fold-higher RNA copy number/PFU ratios than the corresponding WT ZIKV and DENV-2, respectively (Fig. 1F). The results demonstrated that both chimeric viruses were viable but with reduced infectivity.

To study the effect of chimeric prM-E on the viral infection cycle, we compared the replication kinetics of the WT and chimeric viruses on different cell lines, including mosquito C6/36, hamster BHK-21, nonhuman primate Vero, and human liver Huh7 cells (Fig. 1G). CHV-I replicated slightly more slowly than the WT ZIKV on BHK-21 cells but significantly faster on C6/36, Vero, and Huh7 cells. The replication kinetics of CHV-I more closely resembled that of WT DENV-2 on C6/36, Vero, and Huh7 cells, suggesting that replacement of ZIKV prM-E with DENV-2 prM-E converts the cell tropism of CHV-1 to resemble that of DENV-2. In contrast, CHV-II propagated much more slowly than WT DENV-2 on all of the tested cells, probably due to lower efficiency in virion assembly and/or maturation (see details in Discussion).

### Viral prM-E determines virion stability.

We compared the levels of virion stability of ZIKV, DENV-2, and the two chimeric viruses at different temperatures. Consistent with a previous report by Kostyuchenko and colleagues (12), incubation at 37° or 40°C for 30 to 60 min reduced the infectivity of DENV-2 by up to approximately 90%; in contrast, the same treatment decreased the infectivity of ZIKV by <25% (Fig. 1H). Remarkably, the exchange of prM-E from ZIKV with that of DENV-2 in CHV-I destabilized the chimeric virus, whereas swapping the prM-E from DENV-2 with that of ZIKV in CHV-II significantly stabilized the chimeric virus (Fig. 1H). However, it should be noted that the CHV-II did not completely restore the thermostability to the level of WT ZIKV. Collectively, the results demonstrate that prM-E structural proteins are the major determinants of viral thermostability.

### Depletion of the hydrogen bonds around the 5-fold vertex structure of E protein does not affect ZIKV thermostability.

**FIG 2:**
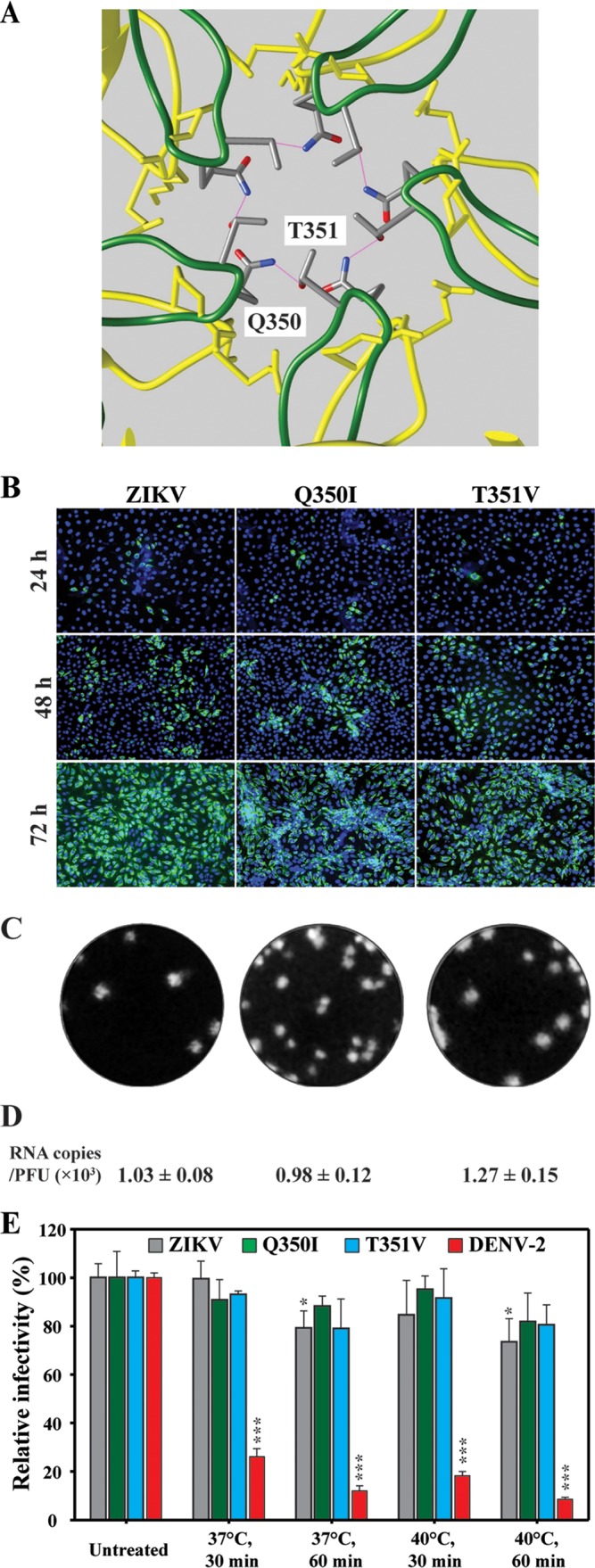
Characterization of ZIKV Q350I and T351V mutants. (A) Structural comparison of ZIKV (PDB identifier [ID] 5iz7) (green) and DENV-2 (PDB ID 3j27) (blue) CD loops around the 5-fold vertex of the virion. Residues Q350 and T351, creating a hydrogen-bond network, are indicated. (B) IFA. Vero cells transfected with ZIKV WT or mutant (Q350I, T351V) RNAs were examined by IFA for E protein expression. (C) Plaque morphologies. Plaques were developed on a Vero cell monolayer after 4.5 days of infection. (D) Numbers of RNA copies/PFU ratios. The RNA copies/PFU ratios calculated at 84 h p.t. are shown. (E) Comparative levels of thermostability of ZIKV Q350I and T351V mutant viruses. One-way ANOVA was performed to analyze the statistical differences between each treatment group and the corresponding untreated group.

Structural comparisons between DENV and ZIKV showed that (i) the CD loop of E protein domain III from ZIKV is more extended than that of DENV around the 5-fold vertex (colored in green and yellow, respectively, in Fig. 2A) and (ii) the extended CD loops of neighboring E molecules in ZIKV form hydrogen-bond interactions through amino acids Q350 and T351 (Fig. 2A). The Q350/T351 interaction was hypothesized to increase the stability of ZIKV (12). Using an infectious cDNA clone of ZIKV, we engineered two mutant viruses to evaluate the functional significance of the Q350/T351 interaction. Mutant Q350I substituted the glutamine at position 350 of the E protein with isoleucine, and mutant T351V substituted the threonine at position 351 with valine. Isoleucine and valine were chosen for the mutagenesis to abolish the hydrogen bond interaction while retaining similar side chains. As shown in Fig. 2B, Q350I and T351V ZIKVs could be recovered from the genomic RNA-transfected Vero cells, as indicated by the increasing number of E-positive cells (Fig. 2B). Mutant Q350I produced plaques slightly smaller than those of the WT virus, while mutant T351 generated plaques similar to those of the WT (Fig. 2C). Comparable RNA copy number/PFU ratios were obtained for WT, Q350I, and T351V viruses (Fig. 2D), suggesting that neither of the mutations affected virion assembly/maturation.

Next, we compared the levels of thermostability of mutants Q350I and T351V with that of WT ZIKV. After incubation at 37° or 40°C for 30 or 60 min, Q350I, T351V, and WT ZIKV retained similar levels of infectivity (Fig. 2E). Our results are in agreement with a recent mutagenesis study using a VLP system (16). Collectively, the data indicate that the hydrogen-bond interaction between Q350 and T351 is not responsible for the thermostability of ZIKV in cell culture.

### The extended CD loop in E domain III is essential for ZIKV assembly.

**FIG 3:**
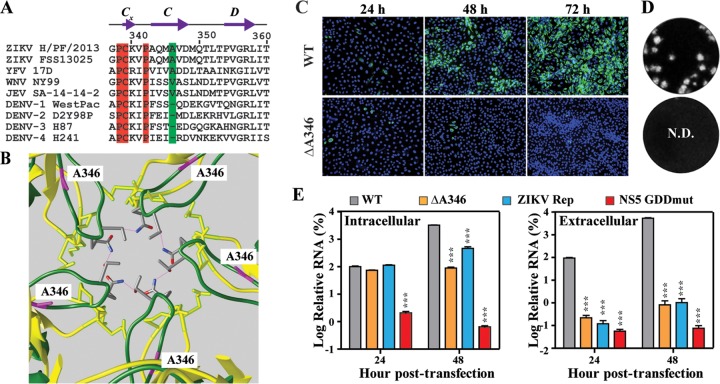
Functional analysis of E protein residue A346 in ZIKV replication. (A) Amino acid sequence alignment. The sequences of the CD loop region (positions 338 to 360) in the E protein domain III were compared among two ZIKV strains (strains H/PF/2013 and FSS13025), YFV strain 17D, WNV strain NY99, JEV strain SA-14-14-2, DENV-1 strain WestPac, DENV-2 strain D2Y98P, DENV-3 strain H87, and DENV-4 strain H241. Residue A346 is highlighted in green. Identical residues are highlighted in red. (B) Location of A346 in the CD loop of ZIKV. Residue A346, which extends the CD loop toward the 5-fold vertex, is colored in purple. (C) IFA analysis of E protein expression in Vero cells transfected with WT and ΔA346 ZIKV RNAs. (D) Plaque morphologies. Plaques were developed on Vero cells after 4.5 days of infection. N.D., not detectable. (E) Effects of ΔA346 mutation on ZIKV replication. At 24 or 48 h p.t., the levels of intracellular and extracellular viral RNA were measured by quantitative RT-PCR. Results of transfection of replicon RNAs (ZIKV Rep) or an NS5 polymerase-defective genomic RNA (NS5 GDDmut) were used as controls. Relative RNA levels were calculated by normalizing the intracellular or extracellular viral RNA levels from each viral RNA-transfected cells to those of WT ZIKV full-length RNA-transfected cells. Each data point represents the mean from results of three independent experiments. At each time point, one-way ANOVA was performed to analyze the statistic differences between WT and mutant RNAs.

Sequence alignment identified an extra amino acid at the C strand of E domain III from several encephalitic flaviviruses (in comparison with the four serotypes of DENV; Fig. 3A). In the case of ZIKV, this extra amino acid, A346, is responsible for the extension of the CD loop (Fig. 3B). To examine the role of the extended CD loop in viral replication, we prepared a ΔA346 mutant ZIKV in which residue A346 was deleted. After transfection of ΔA346 RNA into Vero cells, the number of E-positive cells decreased from 24 to 72 h p.t. (Fig. 3C). No infectious virus could be detected in the culture fluids harvested from the ΔA346 RNA-transfected cells over 5 days p.t. (Fig. 3D). Quantitative reverse transcription-PCR (qRT-PCR) indicated that ΔA346 RNA did replicate in the transfected cells (Fig. 3E, left panel); however, comparable extracellular levels of viral RNA were detected from the cells transfected with ΔA346 RNA, a replicon RNA, or NS5 polymerase-inactive mutant RNA (NS5 GDDmut; Fig. 3E, right panel). These results indicate that deletion of A346 abolished infectious virus production through blocking virion assembly and/or release.

### Virulence of chimeric viruses and ZIKV variants in A129 mice.

**FIG 4:**
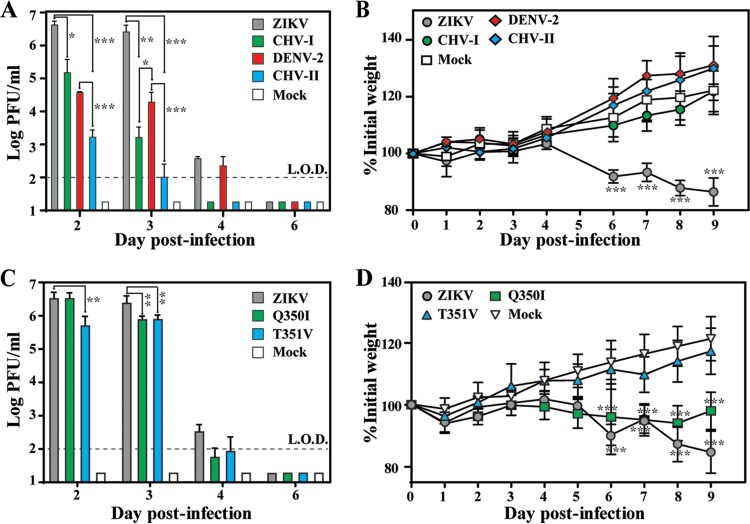
Comparison of levels of virulence of WT and mutant viruses in A129 mice. (A) Mouse viremia after infection with WT or chimeric viruses. Mice (5 mice per group) were intraperitoneally infected with 10^4^ PFU of ZIKV, CHV-I, DENV-2, or CHV-II or were mock infected. The limit of detection (L.O.D.) for viremia was 100 PFU/ml. Each data point represents the mean level of viremia from 2 to 3 mice. At each time point, one-way ANOVA was performed to analyze the statistical significance of viremia differences between ZIKV and CHV-I/CHV-II or between DENV-2 and CHV-I/CHV-II. (B) Mock-infected or infected mice (*n* = 5 per group) were monitored for weight loss over the course of 9 days p.i. of WT or chimeric viruses. Two-way ANOVA was performed to evaluate the statistical significance of weight differences among chimeric virus-infected, WT ZIKV-infected, WT DENV-2-infected, and mock-infected mice at each point. (C) Mouse viremia caused by WT ZIKV and variants (Q350I and T351V). Means and standard deviations (*n* = 4) are shown. (D) Mouse weight postinfection by ZIKV WT and variants (*n* = 8).

A129 mice (lacking interferon α/β receptors) are susceptible to DENV-2 (17) and ZIKV infection (18). This mouse model allowed us to compare the levels of virulence among WT ZIKV, mutant ZIKV (Q350I and T351V), WT DENV-2, and chimeric viruses (CHV-I and CHV-II). Equal amounts (1 × 10^4^ PFU) of each virus were inoculated into 4-week-old mice via the intraperitoneal (i.p.) route. The infected mice were monitored for illness and weight loss, and blood was taken to determine levels of viremia. Mice infected with WT ZIKV showed a peak mean viremia level of 5 × 10^6^ PFU/ml on day 2 postinfection (p.i.) (Fig. 4A) and significant weight loss (Fig. 4B). In contrast, CHV-I generated viremia at a level 1 to 3 log_10_ lower than that seen with WT ZIKV on day 2 to day 3 p.i. (Fig. 4A); moreover, CHV-I infection did not cause significant weight loss (Fig. 4B). No infected mice died, even in the WT ZIKV-infected group. The results suggest that the replacement of prM-E from ZIKV with that of DENV-2 significantly reduced virulence in A129 mice.

Mice infected with WT DENV-2 produced a peak viremia level of 4 × 10^4^ PFU/ml on day 2 p.i. (Fig. 4A), suggesting productive replication in A129 mice. Compared with WT DENV-2, CHV-II showed a >1 to 2 log_10_ lower level of viremia (Fig. 4A). Neither WT DENV-2 nor CHV-II caused any death or weight loss of the infected mice (Fig. 4B). Collectively, these results demonstrate that both CHV-I and CHV-II viruses are attenuated in mice.

Mice infected with ZIKV variant Q350I generated peak viremia comparable to that seen with WT ZIKV on day 2 p.i. and a level of viremia 2-fold lower than that seen with WT ZIKV on day 3 (Fig. 4C). Consistent with the viremia results, the Q350I mutant caused significant weight loss, albeit to a lesser extent than WT ZIKV (Fig. 4D). In contrast, the T351V mutant produced about 8- and 3-fold less viremia than WT ZIKV on day 2 and 3 p.i., respectively (Fig. 4C), with no significant weight loss (Fig. 4D). These data suggest that the T351V mutation, but not Q350I, attenuates ZIKV in A129 mice.

### Mice vaccinated with CHV-I and CHV-II are protected from WT DENV-2 and ZIKV challenge, respectively.

**FIG 5:**
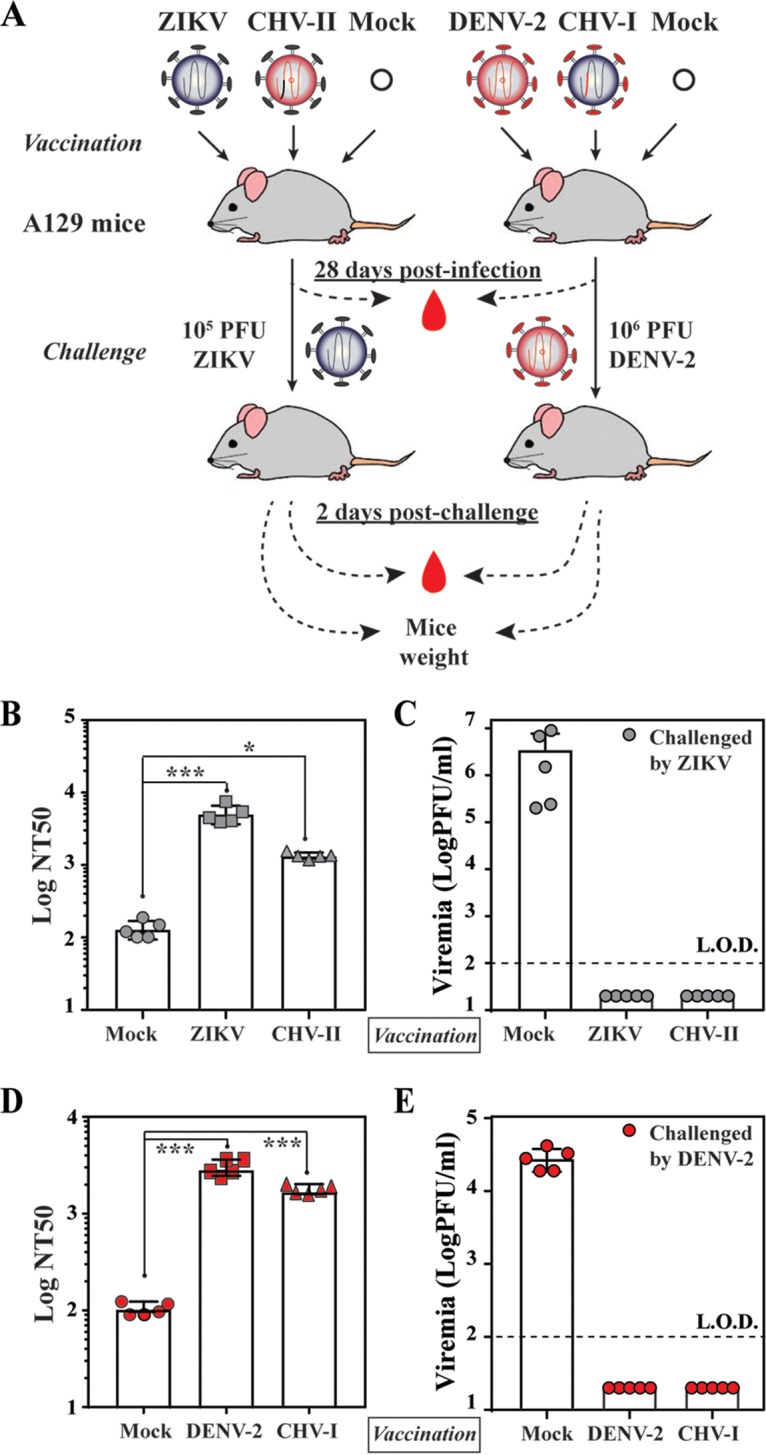
*In vivo* analyses of immunogenicity and protection of chimeric viruses (CHV-I and CHV-II). (A) Experimental scheme. A129 mice (4 weeks old) were infected with 1 × 10^4^ PFU of the indicated viruses via the intraperitoneal route. On day 28 p.i., the mice were challenged with WT ZIKV (1 × 10_5_ PFU) or DENV-2 strain D2Y98P (1 × 10_6_ PFU) intraperitoneally. Mice were bled to monitor prechallenge neutralization titers and postchallenge viremia. (B) NT_50_ against ZIKV. The average of NT_50_ values and standard deviations of results from five mice per group are shown. One-way ANOVA was used to determine significant differences in NT_50_ between mock-vaccinated and ZIKV- or CHV-II-vaccinated mice. (C) Viremia after ZIKV challenge. The average viremia titers and standard deviations (on day 2 postchallenge) of results from five mice per group are shown. The limit of detection (LOD) of viremia was 100 PFU/ml. (D) NT_50_ against DENV-2. The averages of NT_50_ values and standard deviations of results from five mice per group are shown. (E) Viremia after DENV-2 challenge. The mean viremia titers and standard deviations (on day 2 postchallenge) of results from five mice per group are shown.

To examine whether CHV-I and CHV-II could serve as live attenuated vaccines for protection from WT virus infection, 4 weeks after vaccination of mice with these chimeric viruses, we challenged the mice with a high dose of DENV-2 strain D2Y98P (1 × 10^6^ PFU) and ZIKV strain FSS13025 (1 × 10^5^ PFU), respectively. As outlined in Fig. 5A, mice initially immunized with ZIKV or CHV-II or subjected to mock vaccination were challenged with WT ZIKV; alternatively, mice initially vaccinated with DENV-2 or CHV-I or subjected to mock vaccination were challenged with DENV-2. Mice vaccinated with ZIKV or CHV-II produced a prechallenge 50% neutralization titer (NT_50_) of 5.1 × 10^3^ or 1.4 × 10^3^ against ZIKV, respectively (Fig. 5B), and were fully protected from ZIKV challenge (Fig. 5C). Similarly, mice vaccinated with DENV-2 or CHV-I generated a prechallenge neutralization titer of 3 × 10^3^ or 1.8 × 10^3^ against DENV-2, respectively (Fig. 5D), leading to full protection against DENV-2 challenge (Fig. 5E). Collectively, the data suggest that CHV-I and CHV-II have potential for development as live attenuated vaccines against DENV-2 and ZIKV infection, respectively.

## DISCUSSION

Although sequence and structural comparisons showed that ZIKV proteins have similarity to those of other flaviviruses (12, 13), only ZIKV has been associated with congenital diseases (9). The molecular elements responsible for ZIKV pathobiology are currently unknown. The present study aimed to analyze the contribution of ZIKV E protein to virion thermostability and its effect on virulence *in vivo*. Using a reverse genetic approach, we prepared two chimeric viruses by swapping the prM-E genes between DENV-2 and ZIKV in the context of full-length cDNA clones. Comparison of RNA copy number/PFU ratios among different recombinant viruses suggested that WT DENV-2 is >10-fold less infectious than WT ZIKV (Fig. 1F). In addition, the RNA copy number/PFU ratios of prM-E chimeric viruses were 3- to 6-fold higher than those of the corresponding parental backbone viruses (Fig. 1F). The increase in RNA copy number/PFU ratios for CHIV-I and CHV-II could be due to compromised compatibility between prM-E and heterologous viral proteins (including C and all nonstructural proteins) during virion assembly and/or release, leading to the production of progeny viruses with lower specific infectivity. It is well documented that flavivirus assembly is modulated by nonstructural proteins such as NS1 (19), NS2A (20), and NS3 (21). For example, the signal peptide at the C terminus of the C protein regulates flavivirus packaging through coordination of sequential cleavages at the N terminus (by viral NS2B/NS3 protease in the cytoplasm) and C terminus (by host signalase in the endoplasmic reticulum [ER] lumen) of the signal peptide sequence (22). Indeed, we found that substitution of the prM-E from DENV-2 with that of ZIKV, without exchange of the upstream signal peptide sequence (in the backbone of DENV-2), failed to produce infectious chimeric viruses (data not shown).

CHV-I harboring DENV-2 prM-E showed lower infectivity than WT ZIKV (discussed above) but displayed faster replication kinetics on C6/36, Vero, and Huh7 cells (but not on BHK-21 cells). ZIKV is reported to bind to cell surface TAM (Tyro3, Axl, and Mer) receptors but not TIM (T cell immunoglobulin and mucin) phosphatidylserine transmembrane receptor (23), whereas DENV uses both TAM and TIM receptors for entry (24). Substitution of prM-E from ZIKV to DENV-2 might enable CHV-I binding to both TAM and TIM during entry, leading to enhanced replication kinetics. The abundance of TAM and TIM receptors on different cell types may explain the differences in replication kinetics of ZIKV, DENV, and chimeric viruses. Along the same lines, the attenuation of CHV-I in A129 mice (Fig. 3A and B) may also be due to a change in cell tropism *in vivo*. Future studies are needed to compare the virus distributions in different tissues among animals infected with ZIKV, CHV-I, and DENV-2.

Structural comparison between DENV and ZIKV revealed distinct features that might confer higher thermostability to ZIKV and, consequently, its unique pathobiology (12). We performed three sets of experiments to test this hypothesis. First, analysis of chimeric viruses demonstrated that exchange of prM-E genes between DENV-2 and ZIKV could alter the thermostability of chimeras (Fig. 1H), confirming that prM-E proteins are the major determinants of thermostability. Second, our mutagenesis results indicated that the extended CD loop in domain III of ZIKV E protein plays an essential role in virion assembly. Compared with DENV, the extended CD loop is contributed by a 1-amino-acid (A346) insertion into the ZIKV E protein. Recombinant ZIKV containing the A346 deletion retained viral RNA synthesis but abolished virion assembly/release (Fig. 3). A possible explanation is that deletion of A346 shortens the CD loop in domain III of the ZIKV E protein that is required for virion assembly and/or release. Third, our genetic data indicate that the hydrogen-bond interaction between Q350 and T351 around the 5-fold vertex structure is not essential for virion thermostability (Fig. 2E). The result is consistent with a recent mutagenesis study performed using the VLP system (16). Since this VLP system did not allow *in vivo* virulence analysis, we examined the effect of Q350 or T351 mutation on virulence in the A129 mouse model. Interestingly, the virulence of mutant T351V was significantly attenuated in A129 mice, whereas mutant Q350I retained WT virulence (Fig. 4C and D). It should be pointed out that these results do not establish a correlation between thermostability and virulence, because T351V did not change the stability of the virus even though the mutation had an attenuating effect. The T351V mutation must exert its attenuation through an unknown mechanism other than thermostability.

One result that was of practical importance in our study was that it demonstrated the feasibility of generating chimeric ZIKV and DENV-2 with DENV-2 and ZIKV prM-E genes, represented by CHV-I and CHV-II, respectively. Both CHV-I and CHV-II could replicate to high titers of >1 × 10^5^ on Vero cells (a cell line approved for vaccine production; Fig. 1G). The chimeric viruses were highly attenuated in A129 mice, as indicated by decreased viremia and/or weight loss (Fig. 4). Immunization with the chimeric viruses elicited robust antibody (Ab) responses, leading to full protection from WT virus challenge (Fig. 5). These results indicate that the chimeric viruses could be further developed as potential live attenuated vaccines. Exactly the same chimeric approach could be used to engineer ZIKV prM-E into the DENV vaccines currently in phase III clinical trials (25), resulting in DENV-based recombinant ZIKV vaccine.

In summary, we have demonstrated experimentally that the prM-E genes are the major determinants of ZIKV and DENV-2 thermostability. The hydrogen-bond interaction between Q350 and T351 in the CD loop of the ZIKV E protein is not required for virion thermostability, whereas the conformation of the extended CD loop is essential for virus assembly and/or release. The two chimeric viruses (CHV-I and CHV-II) could serve as a starting point for future investigation of ZIKV and DENV pathobiology and development of chimeric vaccines.

## MATERIALS AND METHODS

### Cell culture and antibodies.

BHK-21 and Vero cells were purchased from the American Type Culture Collection (ATCC; Bethesda, MD) and maintained in high-glucose Dulbecco’s modified Eagle’s medium (DMEM) supplemented with 10% fetal bovine serum (FBS) (HyClone Laboratories, South Logan, UT) and 1% penicillin/streptomycin at 37°C with 5% CO_2_. *Aedes albopictus* C6/36 cells were grown in RPMI 1640 containing 10% FBS and 1% penicillin/streptomycin at 30°C with 5% CO_2_. Huh7 cells were maintained in high-glucose DMEM supplemented with 10% fetal bovine serum (FBS), 1% penicillin-streptomycin, and 1% nonessential amino acids (NEAA) at 37°C with 5% CO_2_. All culture medium, NEAA, and antibiotics were purchased from Thermo (Fisher) Scientific. A 4G2 mouse monoclonal antibody (MAb) cross-reactive with flavivirus E protein (ATCC) and goat anti-mouse IgGs conjugated with Alexa Fluor 488 or Alexa Fluor 568 (Thermo Fisher Scientific, Waltham, MA) were used in this study.

### Plasmid construction.

Two infectious clones (ZIKV and DENV-2) were used to construct the prM-E chimeras. The ZIKV infectious clone contains the cDNA sequence of Cambodian strain FSS13025 (18). The DENV-2 infectious clone contains the cDNA sequence of DENV-2 strain D2Y98P (26). For *in vitro* transcription of the genome-length RNAs, a T7 promoter and a hepatitis delta virus ribozyme (HDVr) sequence were placed at the 5′ and 3′ ends of the virus genome in both infectious clones (Fig. 1A), respectively. For construction of CHV-I, two fragments (Fa and Fb) were initially amplified by overlap PCR. Primer sets ZV-NotI-F/ZV-P1-R and ZV-P1-F/ZV-P2-R were used to amplify the Fa fragment. The Fa fragment contains a unique restriction enzyme site (NotI) followed by a gene cassette that includes the T7 promoter, ZIKV 5′ UTR, the codons encoding the ZIKV capsid and anchor C (ZV-C_18_) fused in-frame with the prM and the N-terminal part of E protein of DENV-2, and a BsrGI restriction enzyme site. The Fb fragment was obtained by overlap PCR using primer pairs ZV-P2-F/ZV-P3-R and ZV-P3-F/ZV-3881-R. Fb contains a BsrGI restriction enzyme site, the cDNA sequence of the C-terminal part of DENV-2 E protein, the codons of ZIKV NS1 and NS2A, and a unique restriction enzyme site, SphI. Fragments Fa and Fb were digested with NotI/BsrGI and BsrGI/SphI, respectively, and cloned into the ZIKV infectious clone using NotI and SphI and the three-fragment ligation approach, resulting in the plasmid CHV-I.

A similar approach was used for constructing plasmid CHV-II. Two cDNA fragments (Fc and Fd) were prepared prior to ligation. The Fc fragment was obtained by overlap PCR using primer pairs D2-SacII-F/D2-P1-R and D2-P1-F/D2-P2-R. Fc contained a gene cassette flanked by restriction enzyme sites SacII and SacI. This gene cassette included a T7 promoter, the DENV-2 5′ UTR, and gene codons encoding DENV-2 capsid and ZIKV anchor C (ZV-C_18_) followed by prM and the N-terminal part of E protein from ZIKV. Fd was amplified by overlap PCR using primer pairs D2-P2-F/D2-P3-R and D2-P3-F/D2-SpeI-R. This fragment contained the cDNA sequence of the C-terminal part of ZIKV E protein followed by DENV-2 NS1 and NS2A and by two restriction enzyme sites, SacI and SpeI, at the 5′ end and 3′ end, respectively. The Fc and Fd fragments were digested with SacII/SacI and SacI/SpeI, respectively. The resulting fragments were cloned into the DENV-2 infectious clone, resulting in plasmid CHV-II.

For introduction of mutations into the ZIKV E protein gene, the partial cDNA sequence of ZIKV between AvrII and SphI was initially amplified by PCR using primer pair ZV-NotI-1466F/ZV-3881-R from pFLZIKV. The amplicon was cloned into the pACYC vector through the use of restriction enzyme sites NotI and ClaI, resulting in a pACYC-B shuttle vector. Mutations ΔA346, Q350I, and T351V were individually engineered into shuttle vector pACYC-B by using a QuikChange II XL site-directed mutagenesis kit (Agilent Technologies, Santa Clara, CA). The fragment containing the resulting mutation(s) was digested from the shuttle pACYC-B and cloned into the ZIKV infectious clone through the use of restriction enzyme sites AvrII and SphI. All plasmids were validated by cDNA sequencing. Primer sequences for PCRs are listed in Table 1.

**TABLE 1:** Sequences of PCR primers used in this study

Primer name	Sequence (5′–3′)^a^
D2-SacII-F	GCTTTGCCGCGGCCCTCTCACTTCCCTGTTAAGTAT (SacII)
D2-P1-R	TGAACATCTTGAACAGGAGACGCAGAGGCACAGATACTAG
D2-P1-F	GAACAGGAGACGCAGAGGCACAGATACTAGTGTCGGAATTGTTG
D2-P2-R	CATCTCAGCCTCCAGAGCTCCAGCAA (SacI)
D2-P2-F	GCTGGAGCTCTGGAGGCTGAGATGGAT (SacI)
D2-P3-R	CAACGCAACCACTATCAGCAGAGACGGCTGTGGATAAGAAGAT
D2-P3-F	CAGCCGTCTCTGCTGATAGTGGTTGCGTTGTGAGTTGGAAA
D2-SpeI-R	GAGACTGCAACTAGTAATATTGCATG (SpeI)
ZV-NotI-F	GTCAGCGGCCGCTAATACGACTCACTATA (NotI)
ZV-P1-R	GTGGTTAAATGGAATGCCATGGCTGTGGTCAGCAGGA
ZV-P1-F	GACCACAGCCATGGCATTCCATTTAACCACACGCAACGGAGAA
ZV-P2-R	CTTTCCTGTACACATGGAATATGACATTCCT (BsrGI)
ZV-P2-F	CCATGTGTACAGGAAAGTTCAAAGT (BsrGI)
ZV-P3-R	GCACCCCACATCGGCCTGCACCATAACTCCCAAATACAA
ZV-P3-F	GAGTTATGGTGCAGGCCGATGTGGGGTGCTCGGTGGACTTCTCAAA
ZV-3881-R	CGAATCGATACACGAGGCCAAGGCCAGCAGGCATGC (ClaI, SphI)
ZV-NotI-1466F	TCTGCGGCCGCTAGAGCGAAGGTTGAGATAAC (NotI)

^a^ Restriction enzyme site sequences are underlined.

### RNA *in vitro* transcription and transfection.

Plasmids were linearized and viral RNAs were *in vitro* transcribed as described previously (18, 26). Equal amounts of ZIKV and DENV-2 RNAs were electroporated into Vero cells and BHK-21 cells, respectively, according to protocols described before (18, 26).

### Immunofluorescence assay (IFA).

IFA was performed according to a previously described protocol (18) with modifications. In brief, after fixation and blocking, the cells were incubated with primary antibody 4G2, followed by incubation with goat anti-mouse IgG conjugated with Alexa Fluor 488 or Alexa Fluor 568 as a secondary antibody. Finally, the cells were mounted in a mounting medium with DAPI (4′,6-diamidino-2-phenylindole; Vector Laboratories, Burlingame, CA). Fluorescence images were acquired by the use of a fluorescence microscope equipped with a video documentation system (Olympus).

### Virus replication kinetics and plaque assay.

C6/36 cells (1.2 × 10^6^ cells/well), BHK-21 cells (8 × 10^5^ cells/well), Vero cells (8 × 10^5^ cells/well), or Huh7 cells (8 × 10^5^ cells/well) were seeded into a 6-well plate one day prior to infection. At 16 to 20 h postseeding, cells were infected with equal amounts of ZIKV, DENV-2, or chimeric viruses (at a multiplicity of infection [MOI] of 0.01). Infection was performed in triplicate. After infection at 30°C (C6/36 cells) or 37°C (BHK-21, Vero, and Huh7 cells) for 1 h, virus inocula were removed and cells were washed extensively with phosphate-buffered saline (PBS) to eliminate unbound virus. Afterward, 3 ml of fresh medium was added to each well. From day 1 to day 6 p.i., supernatants were collected daily and clarified by centrifugation prior to storage at −80°C. Virus titers of ZIKV or DENV-2 were determined using a standard cytopathogenic-effect-based plaque assay and Vero cells (18) or BHK-21 cells (27), respectively. Specifically, ZIKV plaques developed on Vero cells were stained by the use of crystal violet after 4.5 days of infection, and DENV-2 plaques formed on BHK-21 cells were stained after 4 days of infection.

### Quantitative reverse transcription-PCR (qRT-PCR).

Viral RNAs in culture fluids were extracted using a QIAamp viral RNA minikit (Qiagen, Valencia, CA), and intracellular total RNAs were isolated using an RNeasy minikit (Qiagen, Valencia, CA). Extracted RNAs were eluted in 50 μl of RNase-free water. Two specific primer/probe sets and one other primer/probe set were designed to specifically target the ZIKV and DENV-2 genomes (Table 2). The probes contained a 5′ 6-carboxyfluorescein (5′-FAM) reporter dye, 3′ Iowa Black FQ quencher (IBFQ), and internal ZEN quencher. All probe-based qRT-PCR assays were performed using a LightCycler 480 system (Roche, Basel, Switzerland) and followed the manufacturer’s protocol by using 15-µl reaction volumes of a QuantiTect Probe RT-PCR kit (Qiagen, Valencia, CA) and 1.5-µl volumes of RNA templates. ZIKV (WT and variants) and CHV-II RNA copy numbers (Fig. 1F and 2D) were determined using ZIKV probe1 with primer set ZIKV_1193F/ZIKV_1269R. DENV-2 WT and CHV-I RNA copy numbers were determined by the use of DENV-2 probe and primer set DENV-2_1463F/DENV-2_1540R (Fig. 1F). The extracellular and intracellular genome lengths or replicon RNA copy numbers were determined using ZIKV probe2 and primer set ZIKV_6878F/ZIKV_6980R (Fig. 2E). RNA transcripts were measured in a range of 10^9^ to 10 copies and were tested as triplicates in parallel to plot a standard curve for estimating the extracellular viral RNA copy numbers. The mRNA level of the housekeeping gene encoding glyceraldehyde-3-phophate dehydrogenase (GAPDH) was measured using an iScript one-step RT-PCR kit with SYBR green (Bio-Rad, Hercules, CA) and primer pair M_GAPDH-F and M_GAPDH-R. All primers were synthesized by Integrated DNA Technologies, Inc. (Coralville, IA). Primer and probe sequences for RT-PCR are listed in Table 2.

**TABLE 2:** Sequence of RT-PCR primers and probes used in this study

Primer name	Sequence (5′–3′)^a^
ZIKV probe1	FAM/AGCCTACCT/ZEN/TGACAAGCAATCAGACACTCAA/3IABkFQ
ZIKV_1193F	CCGCTGCCCAACACAAG
ZIKV_1269R	CCACTAACGTTCTTTTGCAGACAT
ZIKV probe2	56-FAM/TACCGCCAA/ZEN/TGAACTCGGATGGTT/3IABkFQ
ZIKV_6878F	CATGGTAGCAGTGGGTCTTC
ZIKV_6980R	CTCCTCTCTCCTTCCCATTAGA
DENV-2_1463F	CAGGCTATGGCACYGTCACGAT
DENV-2_1540R	CCATYTGCAGCARCACCATCTC
DENV-2 probe	FAM/CTCYCCRAG/ZEN/AACGGGMCTCGACTTCAA/3IABkFQ
M_GAPDH-F	AGGTCGGTGTGAACGGATTTG
M_GAPDH-R	TGTAGACCATGTAGTTGAGGTCA

^a^ FAM, 6-carboxyfluorescein; 56-FAM, 5′ 6-carboxyfluorescein; 3IABkFQ, 3′ Iowa Black FQ quencher.

### Virus thermostability assay.

Equal amounts (2 × 10^5^ PFU/ml) of DENV-2, ZIKV, chimeric viruses (CHV-I and CHV-II), and ZIKV variants (Q350I and T351V) were preincubated for 30 or 60 min at 37°C or 40°C in DMEM medium containing 2% FBS. After treatment, virus titers in each sample were determined by plaque assay. For determinations of the initial input amount of viruses, untreated samples were immediately titrated for plaque assay. The relative infectivity was calculated by normalizing the virus titers of treatment groups to those of untreated groups. Experiments were performed three times in triplicate.

### Mouse experiments.

A129 mice (both genders) were used to examine the virulence of ZIKV, DENV-2, chimeric viruses, and ZIKV variants (Q350I and T351V). Experiments were performed as previously described (17, 18, 28). In brief, 4-week-old A129 mice were infected in cohorts of 5 mice/virus with 1 × 10^4^ PFU via the intraperitoneal route. Calcium- and magnesium-free Dulbecco’s phosphate-buffered saline (DPBS) (Thermo, Fisher Scientific) was used to dilute the virus stocks to the desired concentration. DPBS injection was used as a mock infection. Mice were weighed and monitored daily. On days 2, 3, 4, 6, and 28 postinfection, mice were bled via the retro-orbital (RO) sinus route after being anesthetized. Subsequently, mice were challenged by the use of a high dose of WT ZIKV (10^5^ PFU) or DENV-2 strain D2Y98P (10^6^ PFU) via the intraperitoneal route. Specifically, mice that had initially been mock vaccinated or vaccinated with ZIKV and CHV-II were challenged with ZIKV and mice that had first been immunized with diluent, DENV-2, or CHV-I were challenged with DENV-2. On day 2 postchallenge, mice were bled via the RO sinus route for monitoring viremia. Sera were clarified postcollection by centrifugation at 6,000 × *g* for 5 min and were immediately stored at −80°C prior to plaque assay. Viremia was quantified by plaque assay. Neutralization titers were measured through reporter virus-based methods described below. All animal work was performed in compliance with University of Texas Medical Branch (UTMB) policy as approved by the Institutional Animal Care and Use Committee (IACUC).

### Determination of 50% neutralization titers (NT_50_) using reporter viruses.

A DENV-2 reporter virus containing the *Renilla* luciferase (NGC-Rlu) (29) gene was used to measure the neutralization titers of the mouse serum against DENV-2 in a 96-well plate format. Briefly, Vero cells (1.5 × 10^4^ Vero cells/well) were seeded into a 96-well white opaque plate (Corning Costar) 1 day prior to infection. Mouse sera were initially subjected to 5-fold dilution in phenol red-free DMEM (Thermo, Fisher Scientific) containing 2% FBS, followed by 2-fold (2^1^ to 2^9^) serial dilution. A 5-μl volume of each of the serum dilutions was mixed thoroughly with 45 µl of NGC-Rlu virus (5 × 10^3^ PFU), and each mixture was incubated at 30°C for 1 h to form antibody-virus complexes. Afterward, the serum-virus mixtures were inoculated onto the Vero cell monolayer in a 96-well plate for infection at 30°C for another 2 h. Later, the plate was incubated at 37°C for 48 h. The intracellular luciferase signals were measured using ViviRen substrates (Promega) according to the instructions of the manufacturer. Media containing the same amounts of NGC-Rlu viruses but without mouse serum were used as nontreatment controls. Luciferase signals from the nontreatment controls were set at 100%. Luciferases from each serum dilution-treated samples were normalized to the nontreatment controls.

The NT_50_ of mouse serum against ZIKV was determined using a ZIKV-mCherry reporter (ZIKV-mCh) virus that expresses the mCherry fluorescence protein upon infection of cells. The gene encoding the mCherry fluorescence protein was introduced into the pFLZIKV infectious clone according to a previously described approach (18). The resulting ZIKV-mCh viruses were preincubated with serial dilutions of mouse serum (using the same dilution method as that described above) at 37°C for 1 h. The virus-serum mixtures were further used to infect Vero cells in a 96-well plate. After 48 h postinfection, cells were visualized by fluorescence microscopy using a Cytation 5 cell Imaging Multi-Mode Reader (BioTek) to quantify the mCherry fluorescence-positive cells. The percentage of fluorescence-positive cells in the nontreatment controls was set at 100%. The fluorescence-positive cells from serum-treated wells were normalized to those from nontreatment controls. A four-parameter sigmoidal (logistic) model in GraphPad Prism 7 software was used to calculate the 50% neutralization titers (NT_50_).
